# A Meta-Analysis of the Association between Diabetes Mellitus and Traditional Chinese Medicine Constitution

**DOI:** 10.1155/2021/6390530

**Published:** 2021-08-04

**Authors:** Fan Bai, Hui Luo, Liying Wang, Linghui Zhu, Yuanyuan Guan, Yanfei Zheng, Lingru Li, Qi Wang

**Affiliations:** ^1^Beijing University of Chinese Medicine, Beijing 100029, China; ^2^China Tibetology Research Center, Beijing 100101, China

## Abstract

**Objective:**

To explore the distribution of constitution types of diabetes mellitus (DM) in traditional Chinese medicine (TCM) and to provide evidence-based medicine basis for the prevention and treatment of diabetes.

**Methods:**

PubMed, Embase, Web of Science, and three Chinese databases were searched to include research literature on the relationship between diabetes and TCM constitution. The single rate study of cross-sectional literature was conducted with RStudio software, and the control meta-analysis of the diabetic and nondiabetic population was performed with Review Manager 5.3 software. Two independent reviewers assessed the methodological quality of the studies' data. The main outcomes included the distribution of constitutional types in the diabetic population and the odds ratio (OR) between the two. Effect sizes are expressed as proportions or ORs with 95% confidence intervals (CI).

**Results:**

A total of 28,781 diabetic cases were included in 87 articles. Yin-deficiency, phlegm-dampness, and qi-deficiency accounted for 18% (95% CI (15%, 20%), *P* < 0.01), 17% (95% CI (15%, 19%), *P* < 0.01), and 13% (95% CI (11%, 15%), *P* < 0.01) of the total diabetic cases. The risk of diabetes in people with yin-deficiency and phlegm-dampness was 3.06 (95% CI (1.38–6.78), *P*=0.006) and 1.89 (95%CI (1.05–3.42), *P*=0.03) times higher than that in those with other constitutions, respectively. The distribution of TCM constitution of DM patients varied significantly in different regions and ages.

**Conclusion:**

Yin-deficiency and phlegm-dampness are the common constitution types of diabetic people, and they may also be the risk factors of diabetes. Balanced constitution may be a protective factor of diabetes. More high-quality cohort and case-control studies need to be designed to provide more valuable evidence-based basis for assessing the correlation between DM and TCM constitution.

## 1. Introduction

Diabetes mellitus is a common chronic metabolic disease. With the development of society and the change of people's lifestyles, the incidence of DM has increased year by year, and it has become the most harmful noncommunicable disease on human health after tumor and cardiovascular disease [1]. According to the data released by the International T2DM Federation in 2013, there were 387 million diabetes patients in the world in 2013. China has the largest number of T2DM patients in the world, with 98 million T2DM patients, which is expected to increase to 143 million by 2035 [2]. The high incidence of chronic diseases and the rapid increase in medical costs highlight the importance of public health. In the face of the current high prevalence of diabetes, it is particularly important to carry out effective prevention and treatment measures. Traditional Chinese medicine (TCM) has been used for thousands of years of history as a form of health management, and this health care system has been widely used in many countries of the world [3]. TCM regards that everyone is different from others, so there is no recognized diagnosis and treatment model in the TCM treatment of diabetes, but more emphasis on individual treatment. Different constitution types make individuals suffer from different diseases. Individualized prevention and treatment measures for different types of constitution personalized treatment, so as to achieve a better effect than conventional treatment. The constitution of traditional Chinese medicine is the grasp of “preventive treatment of the disease” [4]. In April 2009, the Constitution in Chinese Medicine Questionnaire (CCMQ) was published and recommended by the China Association of Chinese Medicine as a standard for constitution measurement because of its good reliability and validity [5]. According to the CCMQ, the body constitutions of Chinese population are divided into nine basic types, namely balanced, qi-deficiency, yang-deficiency, yin-deficiency, phlegm-dampness, dampness-heat, blood stasis, qi stagnation, and inherited special. Among them, the balanced constitution is normal, while the other eight are biased and prone to relevant diseases [6]. TCM constitution identification has been widely used in nondisease treatment centers and community health service centers all over the country because of its simple operation, easy to understand, and easy to accept. In recent years, a large number of clinical studies on the relationship between diabetes and TCM constitution have been carried out in various regions, which provides data support for analyzing the distribution law of the TCM constitution of diabetic patients.

This study aims to obtain a larger sample of data on the distribution of TCM constitution types of diabetic patients, to determine the types of constitution prone to diabetes, and to provide evidence-based medical evidence for the effective prevention and treatment of diabetes for “preventive treatment of disease.” This was achieved by a meta-analysis of the current clinical research literature on the TCM constitution of diabetes.

## 2. Materials and Methods

### 2.1. Retrieval Strategy

Clinical studies on the correlation between body constitution and diabetes were searched in PubMed, Embase, Web of Science, China National Knowledge Infrastructure (CNKI), VIP Database (VIP), and Wanfang Database from April 2009 (the CCMQ's publication time) to March 31, 2021. The search terms included “diabetes mellitus,” “tangniaobing” (Chinese pinyin of diabetes), “tizhi” (Chinese pinyin of constitution), and “constitution.” No language, nationality, or publication restrictions were applied. As an example of one specific strategy, the search terms for PubMed were as follows: (diabetes mellitus [Title/Abstract]) AND constitution [Title/Abstract]).

### 2.2. Inclusion/Exclusion Criteria

All clinical literature on the relationship between diabetes and TCM constitution types (including cross-sectional studies, case-control studies, and cohort studies) were included, without limiting the type of research and the form of publication. The details are as follows: (1) Research object: the research object is the diabetic patients with a definite diagnosis; (2) Research tool: the measurement tool used for the constitution identification of the research object is the standard of Classification and Judgment of TCM Constitution issued by the China Institute of TCM in 2009; and (3) the sample size of the study is clear, and the data of the constitution composition are complete.

Exclusion criteria: (1) literature lacking in basic information reports or without statistics of constitution composition; (2) included subjects combined with other systematic serious diseases that may affect their TCM constitution types; (3) the study is limited to a certain kind of constitution population, such as simple phlegm-dampness constitution research; and (4) repeatedly published literature.

### 2.3. Data Extraction and Quality Evaluation on Methodology

Make a data extraction form, including the literature title, the type of research design, the area of the study, the sex of the object of study, the average age, the number of people of each type of constitution, the total sample size, and so on.

The Newcastle-Woodward scale (NOS) [7] was used to assess the methodological quality of the cohort study and case-control study. The scale was compared with 8 items from three aspects: the selection of the study population, the comparability between groups, and the measurement of exposure factors, with a total score of 9 points. The cross-sectional study adopted the standard (referred to as AHRQ standard) recommended by the Agency for Healthcare Research and Quality (AHRQ) [8]. It is divided into 11 items, including data sources, inclusion criteria, observation time, continuity of research objects, subjective factors of evaluators, quality control, and so on, with a total score of 11 points.

### 2.4. Data Analysis

The meta-analysis of the individual rate of each constitution type was carried out by using RStudio-1.1.463 software [9]. Analysis models were chosen by the results of heterogeneity tests. The percentage of 8 different biased constitution types of diabetic people and their 95% confidence interval (CI) were calculated. When the heterogeneity was too large, subgroup analysis was carried out according to regional and age factors, and the inverted funnel chart was used to analyze the degree of publication bias assessed by funnel plot analysis. The Review Manager 5.3 software provided by the Cochrane collaboration network was used to analyze the data synthesis between the diabetic group and nondiabetic group, and the odds ratio (OR) and its 95% CI were used to describe the effect value of a single study. The effect model was selected according to heterogeneity: the fixed effect model was used when heterogeneity was less than 50%, and the random effect model was used when heterogeneity was greater *(I*^*2*^ > 50%).

## 3. Results

### 3.1. Retrieval Process and Results

**Figure 1:**
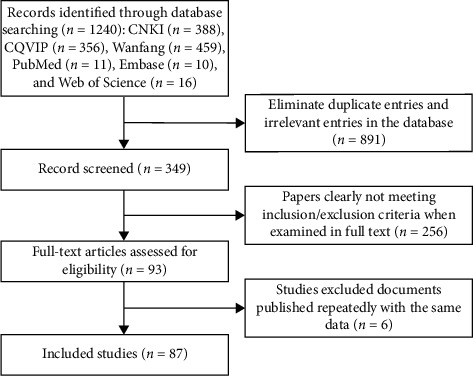
Flow chart of study searching and selection.

Duplicate references were excluded. All titles and abstracts were then screened to eliminate duplicates and obviously irrelevant citations. Following screening of 1240 citations found in the literature search, 93 papers potentially met the inclusion criteria and were examined in full text. 87 studies were included in the final review. The literature screening process and results are shown in Figure 1.

### 3.2. Basic Characteristics and Quality Evaluation of the Inclusion Study

Among all the studies we included, 1 was a Chinese-Malaysian constitution fitness study, and 86 were domestic clinical studies, including 22 provinces, autonomous regions, and municipalities directly under the central government. The study was published in 2009 at the earliest, and the number of studies is increasing year by year. A total of 28,581 patients with diabetes were included in each study, with an average sample size of 329. According to the NOS (9 items) and AHRQ cross-sectional study evaluation criteria (11 items), 7 case-control studies and 80 cross-sectional studies were evaluated. The scores of cross-sectional studies were all 3–7, which were medium- and low-quality literatures, which did not have high research quality and had a high risk of bias. See Table 1 for details.

### 3.3. Meta-Analysis of Biased Constitution Distribution in TCM

According to the number of diabetic patients reported in each study, meta-analysis was carried out. A total of 87 studies reported these data, with a total sample size of 28,581 cases. The results showed that the distribution of 8 biased constitution types in patients with diabetes was yin-deficiency, phlegm-dampness, and qi-deficiency in the highest proportion, and the results of meta-analysis were shown in a forest map. The proportion of the other five types of constitution is less than 10%. The results of the meta-analysis are described in a table.

#### 3.3.1. Yin-Deficiency Constitution

**Figure 2:**
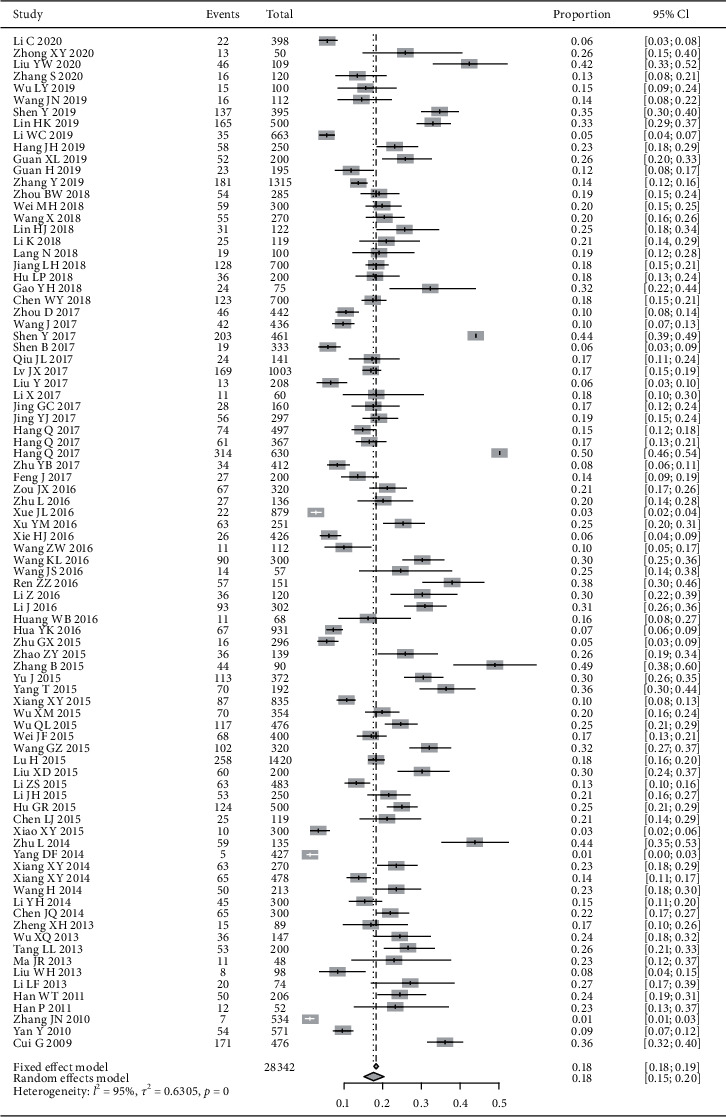
Meta-analysis of the proportion of yin-deficiency constitution in DM patients.

86 studies involving 28,342 cases reported the proportion of yin-deficiency constitution in DM patient population. The random effects model was adopted due to the great heterogeneity of included studies. The results showed that the proportion of yin-deficiency constitution in DM patients was 18% (95% CI (15%, 20%), *P* < 0.01) (Figure 2).

#### 3.3.2. Phlegm-Dampness Constitution

**Figure 3:**
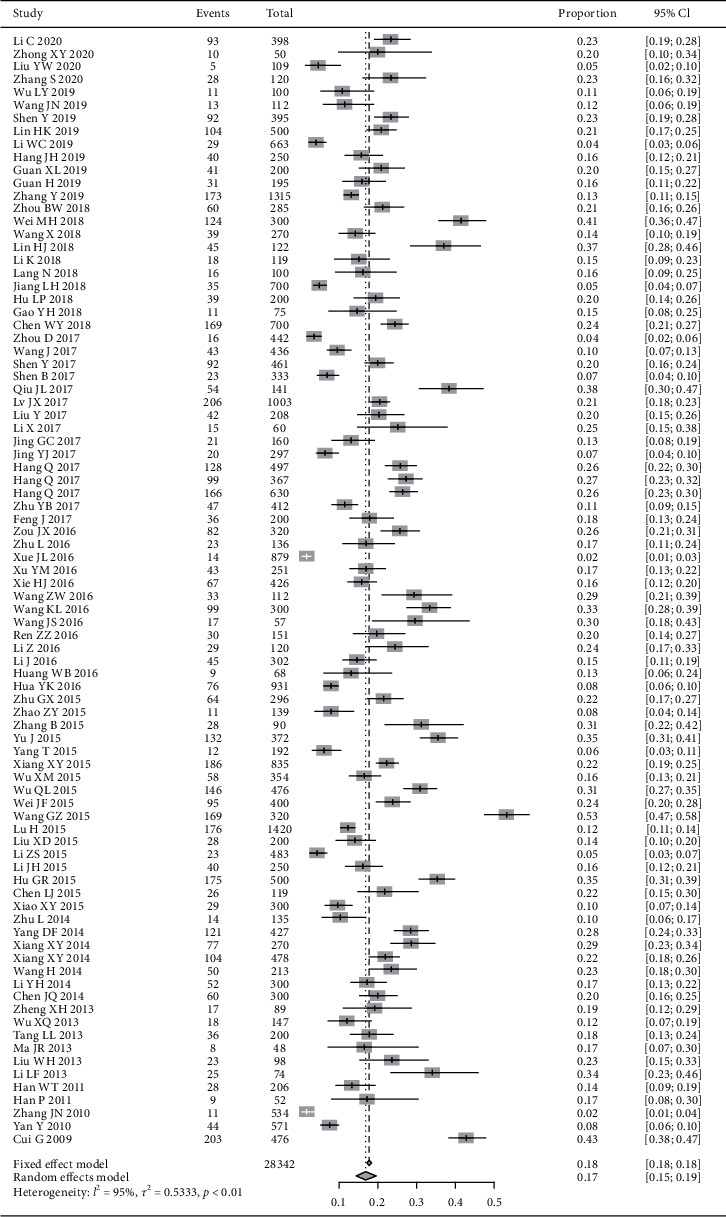
Meta-analysis of the proportion of phlegm-dampness constitution in DM patients.

86 studies involving 28,342 cases reported the proportion of phlegm-dampness constitution in DM patient population. The random effects model was adopted due to the great heterogeneity of included studies. The results showed that the proportion of phlegm-dampness constitution in DM patients was 17% (95% CI (15%, 19%), *P* < 0.01) (Figure 3).

#### 3.3.3. Qi-Deficiency Constitution

**Figure 4:**
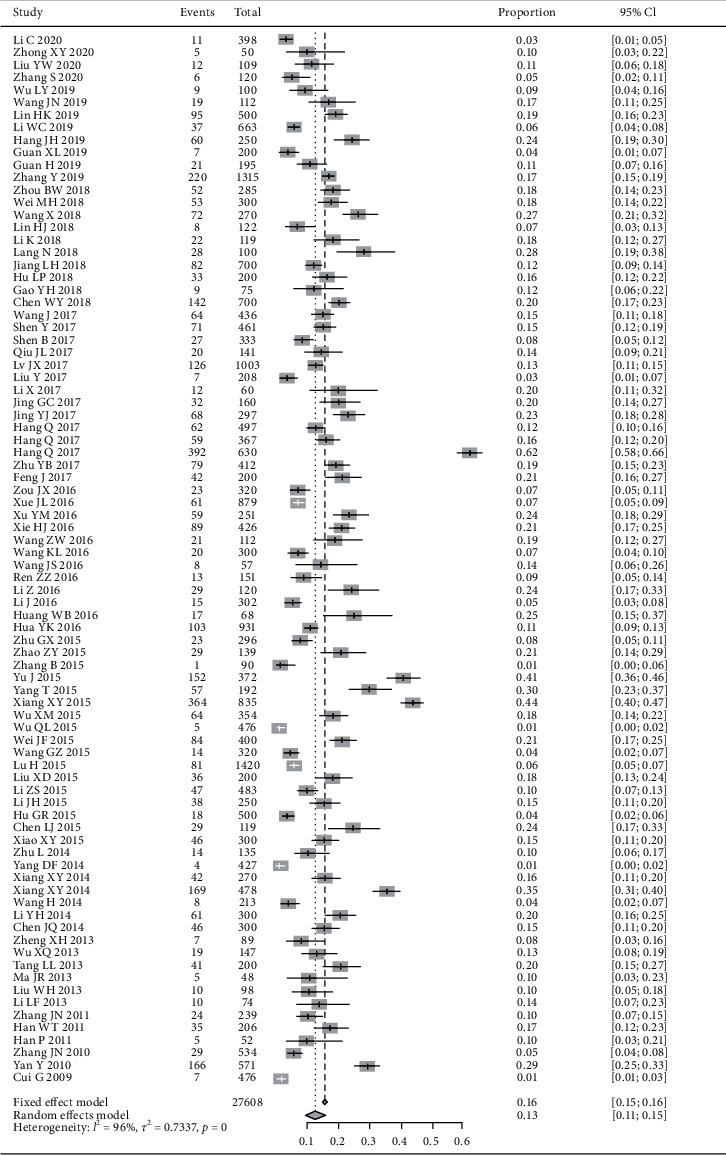
Meta-analysis of the proportion of qi-deficiency constitution in DM patients.

84 studies involving 27,608 cases reported the proportion of qi-deficiency constitution in DM patient population. The random effects model was adopted due to the great heterogeneity of included studies. The results showed that the proportion of qi-deficiency constitution in DM patients was 13% (95% CI (11%, 15%), *P* < 0.01) (Figure 4).

#### 3.3.4. Other TCM Constitutions

The distribution proportion of the other five biased constitution in DM patients is less than 10%, and the order from high to low is yang-deficiency, dampness-heat, blood stasis, qi stagnation, and inherited special constitution. The statistical results show that the *I*^*2*^ values of all types of constitution are more than 50%, suggesting that there is a large heterogeneity, so the random effect model should be used for meta-analysis (Table 2).

### 3.4. Subgroup Analysis of Distribution of TCM Constitution by Region and Age

#### 3.4.1. Region

According to the regions included in the literature, all but one of the reports were conducted in China. Divided by the seven major regions of China, the majority of the literature included in this study is from North China, East China, and South China, with a large sample size, and other regions have smaller literature and smaller sample sizes. Therefore, all the literature was divided into three groups according to North China, East China, and South China to compare the proportions of yin-deficiency, phlegm-dampness, and qi-deficiency reported in the literature from the three regions. The results are shown in Table 3.

#### 3.4.2. Age

According to the average age of participants reported in each study, all the studies were divided into 3 subgroups by average age (≤45, 46–60, and >60). The random effects model was adopted due to the great heterogeneity of included studies. Meta-analysis showed that among the population of DM patients, the distribution of yin-deficiency, phlegm-dampness, and qi-deficiency constitution over 45 years old was significantly higher than that of adults under 45 years old (Table 4).

### 3.5. Meta-Analysis of Distribution of TCM Constitution in DM and General Population

The three most common types of constitution including yin-deficiency, phlegm-dampness, and qi-deficiency were identified in the meta-analysis of the proportion of constitution in DM patients. Then, we further compared it with general population. Seven studies [22, 35, 49, 50, 62, 73, 85] involving 36,546 participants reported the relevant data, so they were included in the meta-analysis.

**Figure 5:**
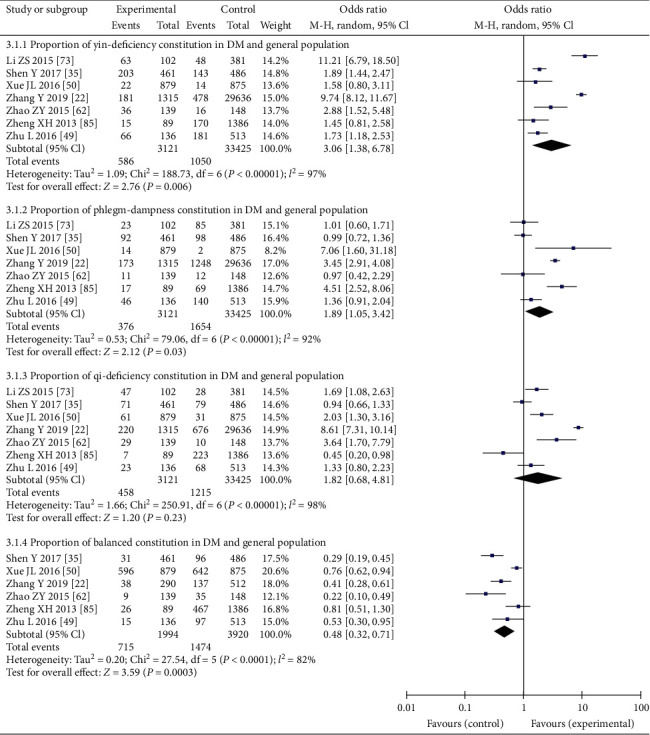
Comparison of distribution of three common traditional Chinese medicine constitutions and balanced constitution between DM patients and general population.

Meta-analysis showed that the OR value of yin-deficiency, phlegm-dampness, and balanced constitution's distribution in DM patients and general population was 3.06 (95%CI: 1.38–6.78), 1.89 (95%CI: 1.05–3.42), and 0.48 (95%CI: 0.32–0.71), respectively. The difference was statistically significant. There was no significant difference in the proportion of qi-deficiency between the DM and general populations, with an OR of 1.82 (95%CI: 0.68–4.81). More details are shown in Figure 5.

### 3.6. Publication Bias

**Figure 6:**
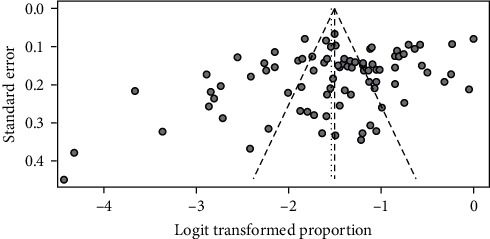
Funnel plot analysis of the distribution of yin-deficiency constitution.

Taking the proportion of yin-deficiency constitution of participants with DM as an index, the inverted funnel diagram was analyzed in the included literature. Considering that TCM constitution research is mostly carried out in different regions and people, and the differences in climate, environment, and lifestyle lead to obvious heterogeneity of each research, so it presents a relatively scattered state, which is related to the particularity of TCM constitution research (Figure 6).

## 4. Discussion

### 4.1. Analysis of the Correlation between DM and the Constitution of TCM

Prevention is the one of the most important essences of TCM, indicating the preventive treatment of diseases. The body constitution in TCM could indicate the patients' overall condition and could form the basis of prevention theory; thus, it has attracted much attention from researchers and clinicians [97]. The TCM constitution refers to the comprehensive, relatively stable, and inherent characteristics of the morphological structure, physiological function, and psychological state formed on the basis of innate endowment and acquired disposition in the course of human life [98]. It can reflect an individual's current health status and future health trends in constitution differences, life processes, psychological condition, and adaptability to natural and social environments. The specificity of individual constitution often causes body's susceptibility to a specific pathogenic factor. For instance, people with phlegm-dampness constitution easily suffer from disease such as diabetes mellitus, metabolic syndrome, and other diseases, with weak adaptability to rainy season and moist environment [99]. The TCM constitution scale provided an objective, generally recognized technical tool and professional standard. Through the constitution classification of TCM, we can determine the individual constitution type of the population and guide the individualized treatment and disease prevention. Correlative studies on constitution and clinical disease have promoted the application of TCM in public health. Relevant research shows that yin-deficiency constitution of hypertension, diabetes, constipation, female menopause, and osteoporosis showed high distribution [100]. This is also consistent with our research results.

A total of 87 articles were included in this study, with a total of 28,781 large samples. It was found that yin-deficiency, phlegm-dampness, and qi-deficiency constitution accounted for 18% (15%–20%), 17% (15%–19%), and 13% (11%–15%) of the total number of people with diabetes, respectively. The results of meta-analysis showed that balanced constitution may be a protective factor of diabetes (OR = 0.48, 95% CI: 0.32–0.71), while yin-deficiency and phlegm-dampness constitution may be the risky factors of it (OR = 3.06, 95% CI: 1.38–2.78 and OR = 1.89, 95% CI: 1.05–3.42).

The constitution distribution of DM patients was significantly different from that of the general population. In 2009, Zhu and Wang published a nationwide cross-sectional study [101] on the constitution of 21,948 general populations in TCM. The results showed that balanced constitution (32.14%) was the most common type of TCM constitution. Qi-deficiency (13.42%), yang-deficiency (9.08%), and phlegm-dampness (9.04%) were common biased constitution. In this study, yin-deficiency constitution was the most common (18%) followed by phlegm-dampness (17%) and qi-deficiency (13%). The proportion of yin-deficiency and phlegm-dampness constitution is much higher than that in general population, while qi-deficiency was close to that of general population. This could illustrate the specificity of constitution characteristics in patients with DM and may also confirm the association between DM and TCM constitution. It is suggested that in the process of prevention and treatment of diabetes in the future, we should identify and focus on the people with yin-deficiency and phlegm-dampness constitution as soon as possible and take corresponding intervention measures in exercise, diet, and so on, to promote the return of biased constitution to balanced constitution.

The results of meta-analysis showed that the proportion of yin-deficiency was the highest in North China followed by South and East China, and the proportion of phlegm-dampness was the highest in South China followed by North China and East China, which may be related to the climatic characteristics of different regions. The climate in North China is cool and dry, the humidity of the air is insufficient, and the body fluid is deficient and dry, so that there are more people with yin-deficiency. The climate in South China is warm and humid, which may easily generate phlegm, dampness, and heat inside the bodies of local residents, which may lead to more people with phlegm-dampness. East China runs through the north and south areas, and the climate contains dry and wet, so people with yin-deficiency and phlegm-dampness account for more. The age subgroup showed that the number of people with diabetes over 45 years old was significantly more than that under 45 years old. Some studies have shown that age is one of the most important factors affecting diabetes [102], advanced age is a risk factor for diabetes, and the prevalence rate of diabetes increases with age. Hence, efforts should be made to adjust the constitution in middle‐aged and elderly populations about diabetes mellitus to reduce the incidence of type 2 diabetes.

### 4.2. Limitations of the Study

From the perspective of the methodology of research design, the literature reports on the basic information related to the research is incomplete, such as the time of inclusion of the research object, basic data, and the original data of each constitution. In the aspect of data statistical analysis, some studies only describe the number and proportion of various constitution types and there is a lack of research on the correlation between constitution types and family heredity, external environment, illness and other factors.

The causes of diabetes are complex, but due to the lack of reported data in the original literature, the analysis angle has some limitations, so it is difficult to deeply analyze the various factors that affect the constitution of people with diabetes. This study is different from other types of meta-analysis. The articles included are mainly observational studies, which can only hint but not prove the causal relationship between disease and constitution. The results of this study need to be confirmed by more high-quality prospective cohort studies and case-control studies. Therefore, there may be a certain risk of deviation in the result.

The included studies are heterogeneous. Due to the large amount of literature, there are significant differences in research time, measurement methods, sample size, age, gender, region, and other aspects. Different from intervention research, individual differences have a great impact on TCM constitution, which is also the characteristic of TCM constitution. Therefore, this study conducted subgroup analyses of the original data by region and age in order to better understand. It is suggested that clinical research methods with higher quality and more standardized contents should be adopted in the future to further explore the relationship between various TCM constitutions and chronic diseases.

### 4.3. Implications for Future Clinical Practice

Referring to the methods of evidence-based medicine in the previous study [103] on the relationship between constitution and disease, this study analyzed the people with high incidence of diabetes from the perspective of TCM constitution. Combined with published studies [103, 104], we have found that phlegm-dampness constitution is closely related to various metabolic diseases, which is of great significance to guide the accurate prevention and treatment of metabolic diseases. In the future clinical practice, we can determine the target of early intervention of diabetes by screening the high-risk groups of yin-deficiency and phlegm-dampness and carry out targeted drug and nondrug intervention according to the constitution and syndrome of TCM, in order to adjust biased constitution and reduce the risk of disease.

## 5. Conclusion

The current systematic review of 87 included studies found that yin-deficiency, phlegm-dampness, and qi-deficiency are the main types of constitution in diabetes patients, of which yin-deficiency and phlegm-dampness are associated with the occurrence of diabetes. They are also risk factors for the incidence of diabetes, while balanced constitution is a protective factor for diabetes. It is suggested that in the future, we should focus on people over 45 years old with yin-deficiency in North China and phlegm-dampness people over 45 years old in South China and increase TCM constitution intervention measures as soon as possible to improve biased constitution and reduce the incidence of diseases. More high-quality observational studies should be designed in the future to provide a more scientific evidence-based basis for the use of personalized diagnosis and treatment of TCM in the prevention and treatment of diabetes.

## Figures and Tables

**Table 1:** Characteristics of included studies.

Study ID	Area	Study design	Sample size	Average age (years)	Gender ratio	Quality evaluation
Li [10]	Jiangxi	CSS	398	75.42 ± 8.65	206/192	5
Zhong and Xue [11]	Guangdong	CSS	50	62.99 ± 3.49	25/25	6
Liu et al. [12]	Henan	CSS	109	57.94 ± 8.69	58/51	4
Zhang et al. [13]	Shanghai	CSS	120	56.88 ± 2.42	65/55	4
Wu et al. [14]	Ningxia	CSS	100	41.6 ± 4.5	68/32	5
Wang et al. [15]	Shanghai	CSS	112	55.8 ± 6.2	54/58	5
Shen et al. [16]	Shanghai	CSS	395	73.81	172/223	4
Lin et al. [17]	Guangdong	CSS	500	61.2 ± 5.3	258/242	6
Li et al. [18]	Guizhou	CSS	663	59.12 ± 6.57	411/252	3
Hang et al. [19]	Guangdong	CSS	250	71.01 ± 5.82	133/117	4
Guan [20]	Jiangsu	CSS	200	U	105/95	3
Guan [21]	Sichuan	CSS	195	69.36 ± 3.41	109/86	7
Zhang et al. [22]	Zhejiang	CCS	1315	U	890/425	6
Zhou [23]	Fujian	CCS	285	56.98 ± 6.75	U	5
Wei and Yang [24]	Guangdong	CSS	300	U	U	5
Wang [25]	Neimongol	CSS	270	66.8 ± 10.5	136/134	6
Lin et al. [26]	Beijing	CSS	122	U	U	4
Li et al. [27]	Beijing	CSS	119	U	71/48	5
Lang [28]	Heilongjiang	CSS	100	75.12 ± 6.34	56/44	7
Jiang et al. [29]	Shanghai	CSS	700	60.14 ± 4.55	350/350	5
Hu et al. [30]	Tianjin	CSS	200	61.32 ± 8.24	96/104	5
Gao et al. [31]	Shandong	CSS	75	69.92	33/42	4
Chen et al. [32]	Zhejiang	CSS	700	74.1 ± 13.1	242/458	5
Zhou et al. [33]	Shanghai	CSS	442	U	187/255	7
Wang [34]	Shandong	CSS	436	58.12 ± 9.63	255/181	7
Shen et al. [35]	Beijing	CCS	461	70.68 ± 10.41	202/259	6
Shen et al. [36]	Beijing	CSS	333	57.80 ± 10.91	133/200	6
Qiu [37]	Xinjiang	CSS	141	61.89 ± 11.37	86/55	5
Lv [38]	Tianjin	CSS	1003	U	U	7
Liu et al. [39]	Jiangxi	CSS	208	52.1 ± 3.4	107/91	5
Li [40]	Beijing	CSS	60	U	31/29	4
Jing et al. [41]	Beijing	CSS	160	56.35 ± 12.82	74/86	4
Jing [42]	Guangxi	CSS	297	57.59 ± 11.12	176/121	5
Huang [43]	Guangdong	CSS	497	67.94 ± 8.64	200/297	4
Huang et al. [44]	Guangdong	CSS	367	67.57 ± 8.77	137/230	4
Huang et al. [45]	Zhejiang	CSS	630	U	236/394	4
Zhu et al. [46]	Nationwide	CSS	412	52.54 ± 13.92	U	7
Feng [47]	Jiangsu	CSS	200	61.4	103/97	7
Zou and Wang [48]	Sichuan	CSS	320	52.11 ± 11.32	167/153	4
Zhu et al. [49]	Beijing	CSS	136	U	U	4
Xue et al. [50]	Shanghai	CCS	879	U	425/454	5
Xu and Liu [51]	Shanxi	CSS	251	59.666	114/133	4
Xie et al. [52]	Henan	CSS	426	U	258/198	5
Wang et al. [53]	Guangdong	CSS	112	86.61 ± 13.39	45/67	5
Wang et al. [54]	Jiangsu	CSS	300	U	144/156	6
Wang [55]	Sichuan	CSS	57	39.04 ± 12.09	10/17	7
Ren et al. [56]	Shandong	CSS	151	63.8	83/68	6
Li [57]	Guangdong	CSS	120	51.9 ± 4.7	68/52	5
Li et al. [58]	Guangxi	CSS	302	65.60 ± 10.96	130/172	6
Huang [59]	Malaysia	CSS	68	64.16 ± 10.03	30/38	5
Hua et al. [60]	Shanghai	CSS	931	73.45 ± 7.37	353/578	4
Zou and Wang [61]	Beijing	CSS	296	49.70 ± 12.28	173/123	4
Zhao [62]	Inner Mongolia	CCS	139	U	84/55	7
Zhang [63]	Henan	CSS	90	40	71/19	5
Yu [64]	Liaoning	CSS	372	60.9 l ± 11.557	173/199	6
Yang [65]	Shandong	CSS	192	63.4 ± 8.0	80/112	7
Xiang and Qian [66]	Jiangsu	CSS	835	63.4	343/492	5
Wu and Hao [67]	Shanxi	CSS	354	U	196/158	5
Wu et al. [68]	Fujian	CSS	476	U	188/288	4
Wei et al. [69]	Shanghai	CSS	400	59.8 ± 11.6	211/189	6
Wang et al. [70]	Guangdong	CSS	320	47.2	175/145	5
Lu [71]	Shanghai	CSS	1420	69.2	574/846	3
Liu et al. [72]	Tianjin	CSS	200	U	U	4
Li [73]	Guangdong	CCS	483	42.03 ± 12.68	263/220	6
Li [74]	Fujian	CSS	250	70.12 ± 11.92	204/261	5
Hu et al. [75]	Jiangxi	CSS	500	57.1 ± 11.4	188/312	6
Chen and Zhou [76]	Zhejiang	CSS	119	57.6	65/54	6
Xiao et al. [77]	Guangdong	CSS	300	65.6 ± 12.6	160/140	5
Zhu et al. [78]	Shandong	CSS	135	55.69 ± 9.87	56/79	6
Yang and Wang [79]	Yunnan	CSS	427	U	U	6
Xiang and Ran [80]	Jiangsu	CSS	270	U	116/154	5
Xiang [81]	Jiangsu	CSS	478	63.1 ± 9.1	201/277	5
Wang et al. [82]	Beijing	CSS	213	56.55 ± 12.14	99/104	6
Li et al. [83]	Zhejiang	CSS	300	56.57 ± 7.91	152/148	5
Chen et al. [84]	Henan	CSS	300	52.12 ± 12.19	152/148	5
Zheng and Jian [85]	Guangdong	CCS	89	U	54/35	6
Wu et al.[86]	Guangdong	CSS	147	U	U	6
Tang [87]	Guangxi	CSS	200	52.78 ± 6.62	99/101	3
Ma [88]	Guangdong	CSS	48	56.9 ± 6.7	35/13	4
Liu [89]	Guangdong	CSS	98	56.6 ± 4.8	55/43	6
Li et al. [90]	Guangdong	CSS	74	62.6	39/35	5
Zhang et al. [91]	Hong Kong	CSS	239	59 ± 10.33	119/120	5
Han [92]	Beijing	CSS	206	65 ± 12	91/115	6
Han et al. [93]	Beijing	CSS	52	58.14 ± 8.2	41/11	4
Zhang [94]	Fujian	CSS	534	57.67 ± 11.17	258/276	5
Yan et al. [95]	Henan	CSS	571	51.69 ± 8.81	284/287	6
Cui [96]	Shanxi	CSS	476	U	188/288	4

CSS, cross-sectional study; CCS, case-control study; U, unclear, indicating no report.

**Table 2:** Meta-analysis of the proportion of other five constitutions in DM patients.

Constitution	Studies	Participants	Proportion (%)	95% CI	*P*	*I*^*2*^(%)
Yang-deficiency	79	26025	8	7%–9%	<0.01	91
Dampness-heat	78	26209	7	6%–9%	<0.01	96
Blood stasis	83	27290	6	5%–7%	<0.01	90
Qi stagnation	73	24692	4	3%–5%	<0.01	90
Inherited special	62	21295	2	1%–2%	<0.01	76

**Table 3:** Meta-analysis of common constitution proportion of DM in three regions.

Constitution	Region	Studies	Number of occurrence	Total sample size	Proportion (%)	95% CI	*P*
Yin-deficiency	East China	32	2472	14386	16	13%–20%	<0.01
	South China	20	944	4793	17	13%–21%	<0.01
	North China	18	961	4575	20	16%–25%	<0.01

Phlegm-dampness	East China	32	2351	14386	15	12%–19%	<0.01
	South China	19	980	4554	20	15%–26%	<0.01
	North China	18	812	4575	17	14%–20%	<0.01

Qi-deficiency	East China	31	2241	13991	11	7%–16%	<0.01
	South China	20	690	4793	13	11%–16%	<0.01
	North China	18	662	4575	13	9%–17%	<0.01

**Table 4:** Meta-analysis of distribution of TCM constitutions by age in DM patients.

Constitution	Age	Studies	Participants	Proportion (%)	95% CI	*P*
Yin-deficiency	≤45	4	136	23	12%–38%	<0.01
	46–60	31	1352	14	11%–19%	<0.01
	>60	29	1916	19	15%–23%	<0.01

Phlegm-dampness	≤45	4	79	15	7%–31%	<0.01
	46–60	30	1356	15	11%–19%	<0.01
	>60	29	1846	18	16%–21%	<0.01

Qi-deficiency	≤45	4	65	8	4%–14%	≤0.01
	46–60	31	1173	12	9%–15%	<0.01
	>60	28	1699	16	13%–20%	<0.01

## Data Availability

The relevant data used to support the results of this study are included in this article.
